# Relative lateral wall thickness is an improved predictor for postoperative lateral wall fracture after trochanteric femoral fracture osteosynthesis

**DOI:** 10.1038/s41598-023-43929-7

**Published:** 2023-10-18

**Authors:** Kenneth P. van Knegsel, C.-E. Hsu, K.-C. Huang, Emir Benca, Torsten Pastor, Bergita Ganse, Peter Varga, Boyko Gueorguiev, Matthias Knobe

**Affiliations:** 1 Department of Orthopedics and Trauma Surgery, Lucerne Cantonal Hospital, Spitalstraβe 16, 6000 Lucerne, Switzerland; 2 AO Research Institute Davos, Clavadelerstrasse 8, 7270 Davos, Switzerland; 3 Medical Faculty, University of Zurich, Raemistrasse 100, 8091 Zurich, Switzerland; 4 Department of Orthopedics, Taichung Veterans General Hospital, Taichung, Taiwan; 5 Sports Recreation and Health Management Continuing Studies-Bachelor’s Degree Completion Program, Tunghai University, Taichung, 407 Taiwan; 6 Department of Orthopedics, Asia University Hospital, Taichung, Taiwan; 7 Department of Occupational Therapy, Asia University, Taichung, Taiwan; 8 Department of Orthopedics and Trauma Surgery, Medical University of Vienna, Währinger Gürtel 18-20, 1090 Vienna, Austria; 9 Werner Siemens Foundation Endowed Chair for Innovative Implant Development, Clinics and Institutes of Surgery, Saarland University, 66421 Homburg, Germany; 10 Department of Trauma, Hand and Reconstructive Surgery, Saarland University Hospital, 66421 Homburg, Germany; 11 Medical Faculty, RWTH Aachen University Hospital, Pauwelsstrasse 30, 52074 Aachen, Germany; 12 Department of Orthopaedic Trauma, Westmuensterland Hospital, Wuellener Strasse 101, 48683 Ahaus, Germany

**Keywords:** Geriatrics, Surgery

## Abstract

Lateral wall thickness is a known predictor for postoperative stability of trochanteric femoral fractures and occurrence of secondary lateral wall fractures. Currently, the AO/OTA classification relies on the absolute lateral wall thickness (aLWT) to distinguish between stable A1.3 and unstable A2.1 fractures that does not take interpersonal patient differences into account. Thus, a more individualized and accurate measure would be favorable. Therefore, we proposed and validated a new patient-specific measure—the relative lateral wall thickness (rLWT)—to consider individualized measures and hypothesized its higher sensitivity and specificity compared with aLWT. First, in 146 pelvic radiographs of patients without a trochanteric femoral fracture, the symmetry of both caput-collum-diaphyseal angle (CCD) and total trochanteric thickness (TTT) was assessed to determine whether the contralateral side can be used for rLWT determination. Then, data of 202 patients were re-evaluated to compare rLWT versus previously published aLWT. Bilateral symmetry was found for both CCD and TTT (p ≥ 0.827), implying that bone morphology and geometry of the contralateral intact side could be used to calculate rLWT. Validation revealed increased accuracy of the rLWT compared with the gold standard aLWT, with increased specificity by 3.5% (Number Needed to Treat = 64 patients) and sensitivity by 1% (Number Needed to Treat = 75 patients). The novel rLWT is a more accurate and individualized predictor of secondary lateral wall fractures compared with the standard aLWT. This study established the threshold of 50.5% rLWT as a reference value for predicting fracture stability in trochanteric femoral fractures.

## Introduction

Trochanteric femoral fractures (TFF) are common in the elderly population and represent a serious problem for both the patient and healthcare system^1,2^. The incidence is estimated at 6.26 million fractures per year by 2050 worldwide^2,3^. Despite advanced treatment options, the 2-year mortality rate after TFF is between 9 and 43%^1,4,5^. Postoperative complications are frequent and include implant failure, surgical site infection, deep vein thrombosis, and secondary lateral wall fracture (sLWF)^1,4–7^. The gold standard treatment of TFF is closed reduction and internal fixation (CRIF), where the correct implant choice and position after appropriate fracture reduction is paramount to minimize the risk of secondary complications^1,5–8^.

For TFF, lateral wall thickness (LWT) was shown to be a strong predictor of postoperative fracture stability^6–8^. A smaller LWT increases the risk of an intra-/postoperative sLWF^6,8^. Postoperative sLWF were reported in 20–30% of the cases treated with a dynamic hip screw (DHS; DePuy Synthes, Zuchwil, Switzerland)^6,8,9^. In case of sLWF, revision surgery is required in 22–45% of cases^6,8^.

In 2018, the AO/OTA classification has been revised regarding TFF stability based on new insights on the importance of the LWT^10^. The original classification assigned TFF into different categories based on fracture morphology contributing to fracture instability, such as comminution, subtrochanteric or femoral neck extension, and trochanter detachment^10–12^. The updated AO/OTA classification considers A2.1 fractures in the original classification as being either stable A1.3 or unstable A2.1 fractures, based on an absolute LWT (aLWT) threshold of 20.5 mm^10,13^. All A2 fractures are considered unstable, but the degree of their instability and therefore the required treatment remain controversial^14^. Consequently, the definition of an unstable A2 fracture pattern must be established so that treatment approaches can be differentiated^12,14^. The aLWT is evaluated in anteroposterior (AP) radiographs and defined as the distance between the fracture line and a lateral point located 3 cm distally of the innominate tubercle, measured at an angle of 135° with respect to the femoral shaft axis^6,10^. Although this is a straightforward method to determine aLWT, it does not consider interpersonal anatomical differences. A given aLWT could lead to a larger or smaller extent of instability depending on the size of the femur. Furthermore, the caput-collum-diaphyseal-angle (CCD) affects the load transmission between the femoral head and shaft and—assuming appropriate reduction—the patient-specific magnitude of this angle should therefore be considered in the analysis of fracture stability. Currently, a constant CCD of 135° is considered when assessing the aLWT^6,10^. Consideration of the patient-specific anatomy could lead to a more accurate fixation stability estimation^15–17^.

The mirrored contralateral femoral anatomy is commonly used for preoperative planning when the ipsilateral femur is fractured. Measurement accuracy relies on the assumption of bilateral femoral symmetry. Several studies supported the theory of proximal femoral bilateral symmetry in patients with normal morphology and confirmed the feasibility of contralateral preoperative planning^18–20^.

Therefore, this study aimed to investigate whether an adapted, patient-specific relative lateral wall thickness (rLWT) measuring protocol using the contralateral femur as a template could allow for higher sensitivity and specificity in sLWF prediction compared to the current standard aLWT.

## Materials and methods

### Study outline

Two sub-studies were performed to develop and validate the novel rLWT measure.

Sub-study 1 was designed to evaluate three important factors in patients without TFF (Dataset 1) before evaluating rLWT in sub-study 2. First, the bilateral symmetry of the parameters required for the rLWT measure was evaluated. Second, the importance of CCD for rLWT was predicted by determining the relationship between CCD and total trochanteric thickness (TTT), and third, the rLWT value corresponding to the previously published aLWT threshold of 20.5 mm was calculated to predict the rLWT threshold.

In sub-study 2, the prediction accuracy of rLWT was compared with the standard aLWT for assessment of TFF stability within a cohort of patients treated with DHS (previous published, Dataset 2), including cases with sLWF^6^.

### Patients

#### Sub-study 1

Dataset 1 consisted of standardized AP pelvic radiographs of 146 adult patients acquired in standing or supine position (age 67.8 ± 17.0 years (mean value ± standard deviation (SD)), range 18–95 years, 69 women and 77 men). These radiographs were retrospectively collected from the database of the Lucerne Cantonal Hospital and anonymized prior to analyses. The local Ethical Committee (Swiss Association of Research Ethics Committees, Req-2021-01202) waived the need for obtained informed consent based on complete anonymization of the data in accordance with the Declaration of Helsinki. Exclusion criteria were signs of previous surgery, injury or disease around the greater trochanter, and a missing reference sphere.

#### Sub-study 2

Dataset 2 consisted of radiographs of AO/OTA 31-A1 and AO/OTA 31-A2 TFF cases treated with a DHS at the Department of Orthopedics, Taichung Veterans General Hospital, Taichung, Taiwan between January 2003 and May 2012. This cohort was identical to that of a previous study investigating aLWT^6^. The study was approved by the Institutional Review Board of Taichung Veterans General Hospital (number TCVGH-CE12183). Written informed consent was collected in accordance with the Declaration of Helsinki. Data between the study sites was shared in an anonymized manner. The rLWT measurements (described below) were performed retrospectively. Exclusion criteria were missing informed consent, non-traumatic fractures, no available intact femur radiograph (ipsi- and contra-lateral), previous fracture in the trochanteric region, osteosynthesis with a technique different from DHS, poor fracture reduction defined as either > 20° angulation on the lateral radiograph or > 4 mm displacement of any fragment, tip-apex distance (TAD) > 25 mm (measured according to the method by Baumgaertner et al.^21^), or a follow-up period shorter than six months^21^. In total, 202 patients were included, 101 females and 101 males (age 77.5 ± 10.36 years, range 33–94 years). All patients received DHS fracture fixation according to the manufacturer's instructions. Postoperative treatment consisted of first mobilization 24–72 h postoperatively with unrestricted weight-bearing under the supervision of a physiotherapist.

### Measurements

#### Sub-study 1

In sub-study 1, non-fractured hip radiographs of Dataset 1 were used to evaluate the CCD and TTT differences between the left and right femur of the same patient and the rLWT value corresponding to the 20.5 mm aLWT threshold^1^. The radiographic images were calibrated with a 25 mm diameter reference sphere. Subsequently, CCD and TTT were measured on both sides by a single surgeon at two different time points to determine the intercorrelation coefficient (ICC), and the average values were used for analysis. TTT was defined as the distance between the lateral cortex of the femur and the intertrochanteric line measured along the caput-collum axis line used for CCD measurement.

#### Sub-study 2

**Figure 1:**
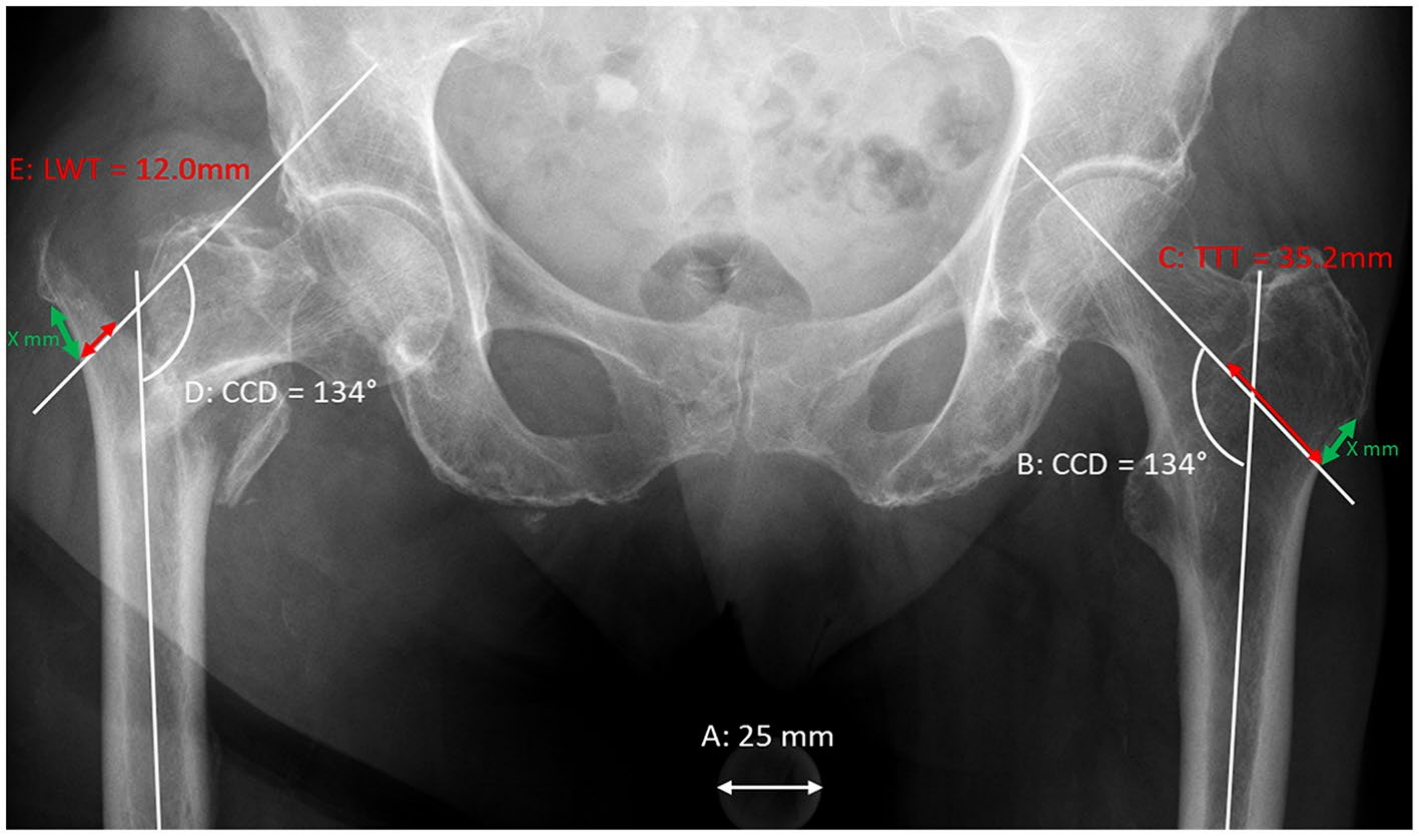
Illustration of the new measurement method for assessment of rLWT. (**A**) Calibration, (**B**) Defining CCD on the healthy contralateral side, (**C**) (red): Defining TTT as the distance from the lateral cortex to the intertrochanteric line along the caput-collum axis line, (**D**) Mirroring the CCD from the contralateral (**B**) to the fractured side and positioning the caput-collum axis line at the same distance to the innominate tubercle (green arrows), (**E**) (red): Measuring the LWT from the lateral cortex to the fracture line along the caput-collum axis line.

In sub-study 2, the new measurement protocol for evaluating rLWT threshold was established (Fig. 1). Two patient-specific dimensions parameters, determined in AP pelvic radiographs, were considered for measurements. CCD was chosen anticipating its influence on load transmission and TTT was considered to represent the femoral dimension. All measurements were performed on calibrated standard AP-view pelvic radiographs of Dataset 2. In seven cases, the contralateral side had a history of a hip fracture, disease, or total hip replacement. For these cases, CCD and TTT were measured on the ipsilateral side using a previous radiograph acquired before fracturing. No pelvic radiographs with an intact femur were present for three patients. For these cases, the contralateral TTT and CCD were measured on an abdominal radiograph. Subsequently, rLWT was calculated as rLWT = (LWT/TTT) × 100% where LWT was measured from the lateral cortex to the fracture line along the caput-collum axis line, considering the contralateral CCD. The distance from the tuberculum vastoadductorium (innominate tubercle) to the lateral cortex on the contralateral side was used for positioning the caput-collum axis line at the ipsilateral side (Fig. 1, green arrows). These measurements were performed by two independent surgeons to determine the ICC, and the average of the two measurements was used. The demographic data age, gender and fracture side of Dataset 2 demonstrated no significant differences between the non-sLWF (n = 166) and sLWF (n = 42) groups in the previous study^6^.

### Statistical analysis

Normality distribution of the data was assumed based on the central limit theorem. Significant difference was set at 95% confidence level. IBM SPSS Statistics 26 (IBM Corp., Armonk, New York, USA) was used.

#### Sub-study 1

In sub-study 1, the differences between the left and right femurs were evaluated with regard to CCD and TTT using Paired-Samples t-test. Moreover, a linear regression analysis was performed to determine the CCD-TTT relationship, and the Pearson's correlation coefficient (r) was calculated. Additionally, the rLWT value corresponding to the previously published aLWT threshold of 20.5 mm was calculated to predict the rLWT threshold^1^.

#### Sub-study 2

In sub-study 2, the rLWT was compared between the sLWF and non-sLWF groups using Independent-Samples t-test. The Receiving Operating Characteristics (ROC) curve was calculated for rLWT and the area under the curve (AUC) was compared with the previously published aLWT results within the same cohort^6^. With regard to sLWF, the Number Needed to Treat was calculated as 1/absolute risk reduction for patients with aLWT < 20.5 mm compared to patients with rLWT < 50.5%, as well as for patients with aLWT > 20.5 mm compared to patients with rLWT > 50.5%.

## Results

### Sub-study 1: intra-patient symmetry (non-fractured Dataset 1)

The results from sub-study 1 (ICC, 0.953) are presented in Table 1. CCD and TTT did not differ significantly between the sides, p ≥ 0.827. Further stratification between men and women demonstrated no significant differences between left and right sides for either CCD or TTT, p ≥ 0.076. There was a moderate, positive correlation between these two variables, r = 0.438, N = 292; p < 0.001. The mean TTT increased by 0.4 mm for each degree increase of CCD. The aLWT threshold value of 20.5 mm corresponded to an rLWT value of of 52.5 ± 6.8%.

**Table 1:** Descriptive data of non-fractured Dataset 1 presented in terms of mean value and SD, including p-values from the comparison of CCD and TTT between the two sides.

Included patients	Age (years)	CCD (°)			p-value	TTT (mm)			p-value
		Total	Left	Right		Total	Left	Right	
All patients (n = 146)	67.8 ± 17.1	129.4 ± 6.4	129.4 ± 6.0	129.5 ± 6.2	0.827	40.0 ± 5.5	39.4 ± 5.7	40.6 ± 5.3	0.992
Men (n = 69)	63.9 ± 17.3	129.4 ± 6.2	129.3 ± 6.2	129.4 ± 6.2	0.967	42.0 ± 5.0	41.6 ± 5.1	42.4 ± 4.8	0.341
Women (n = 77)	71.3 ± 16.1	129.5 ± 5.1	129.4 ± 5.9	129.6 ± 6.0	0.797	38.2 ± 5.4	37.4 ± 4.7	39.0 ± 5.9	0.076

### Sub-study 2: comparison of rLWT and aLWT (Dataset 2)

The results from sub-study 2 (ICC, 0.960) are presented in Table 2. Fourty-two (20.8%) of the 202 patients had sLWF. Patients without sLWF had a significantly larger rLWT (61.8 ± 15.0%) compared to the rLWT (42.7 ± 10.5%) of patients with sLWF, (p < 0.001, 95% confidence interval of the difference (CI) 14.4–24.0). After grouping the patients based on the AO/OTA fracture type, a significantly larger rLWT was seen in patients without sLWF compared to patients with sLWF in both groups with AO/OTA 31-A1 and AO/OTA 31-A2 fractures, p ≤ 0.002. Furthermore, patients with AO/OTA 31-A1 fractures had a significantly greater rLWT compared with AO/OTA 31-A2 fractures (p < 0.001, 95% CI 11.4–19.3).

**Table 2:** Descriptive data of Dataset 2 presented in terms of quantity (n), percentage (%), mean value and SD, including p-values from the comparisons between patients with and without sLWF.

Parameter	All patients	Patients with sLWF	Patients without sLWF	p-value
Included patients (n, %)	202 (100.0)	42 (20.8)	160 (79.2)	–
Patients with AO/OTA 31-A1 fractures (n, %)	93 (46.0)	3 (3.2)	90 (96.8)	–
Patients with AO/OTA 31-A2 fractures (n, %)	109 (54.0)	39 (35.8)	70 (64.2)	–
rLWT among all patients (mean value ± SD, %)	57.8 ± 16.1	42.7 ± 10.5	61.8 ± 15.0	< 0.001 [95% CI 14.4–24.0]
rLWT among patients with AO/OTA 31-A1 fractures (mean value ± SD, %)	66.1 ± 14.6	40.7 ± 9.9	66.9 ± 14.0	0.002 [95% CI 10.0–42.5]
rLWT among patients with AO/OTA 31-A2 fractures (mean value ± SD, %)	50.8 ± 14.0	42.9 ± 10.7	55.3 ± 13.7	< 0.001 [95% CI 7.4–17.4]

**Figure 2:**
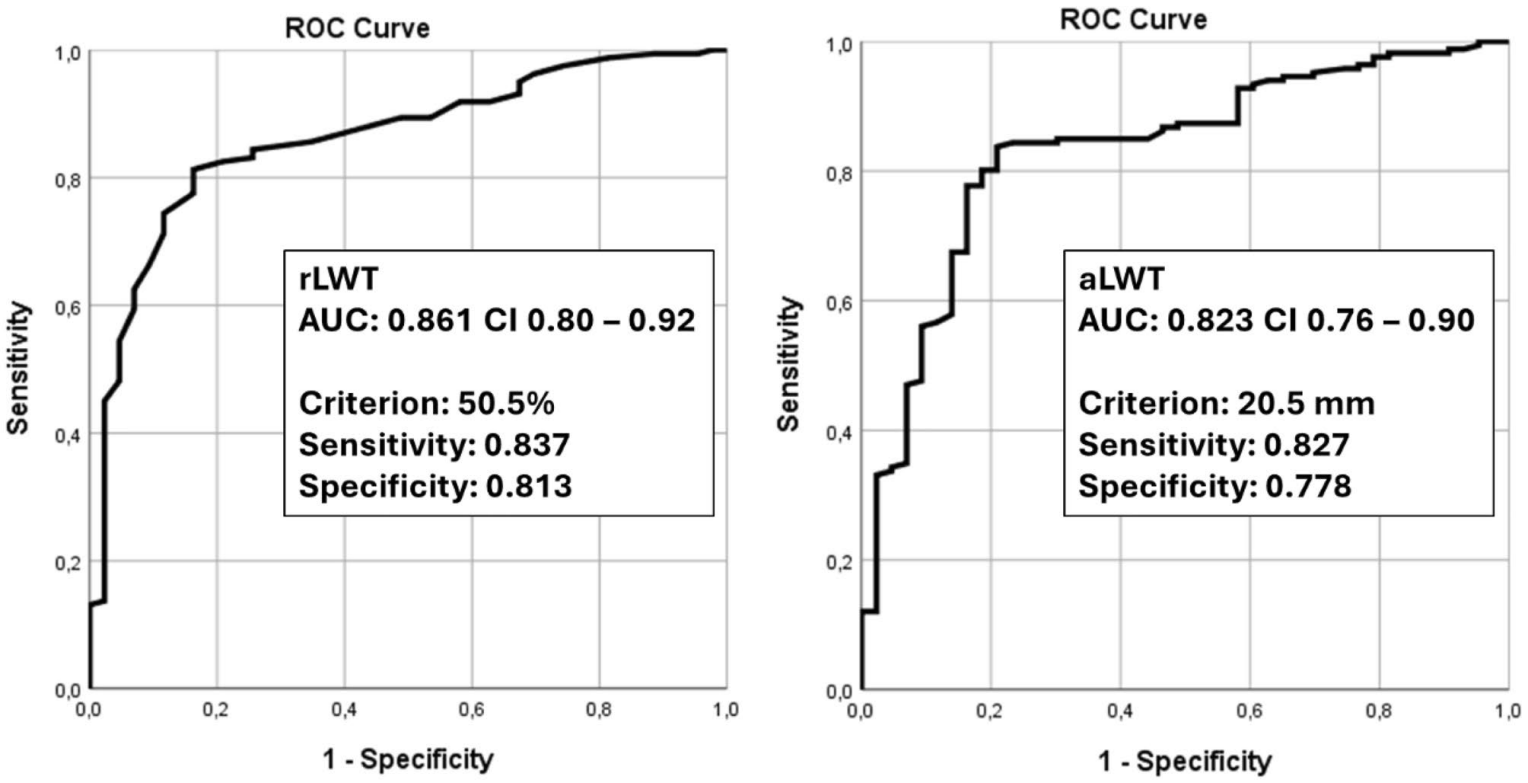
ROC curves with sensitivity plotted against 1—specificity. Left: rLWT (novel measure). Right: aLWT (standard measure).

The ROC analysis revealed an rLWT AUC of 0.861 (95% CI 0.80–0.92, p < 0.001), being higher compared with the aLWT AUC of 0.823 (95% CI 0.76–0.90, p < 0.001) (Fig. 2). The optimal rLWT threshold defined by maximizing the Youden's index (specificity = 83.7% and sensitivity = 81.3%) was 50.5%, which is 2% less than the one predicted in sub-study 1. The specificity and sensitivity were improved for rLWT in comparison to aLWT by 3.5% and 1%, respectively. The Number Needed to Treat for specificity and sensitivity—64 and 75 patients, respectively—was calculated based on the data presented in Table 3 (e.g., treating 64 patients will prevent 1 patient from being categorized as stable while being unstable—subsequently having an sLWF, and treating 75 patients will prevent one patient from being categorized as unstable while being stable).

**Table 3:** Data used for calculation of the Number Needed to Treat in case of predicted unstable and stable TFF based on rLWT and aLWT.

Predicted unstable TFF			Predicted stable TFF		
Parameter and range	Patients without sLWF (n)	Patients with sLWF (n)	Parameter and range	Patients without sLWF (n)	Patients with sLWF (n)
rLWT < 50.5%	30 (a)	35 (c)	rLWT > 50.5%	130 (a)	7 (c)
aLWT < 20.5 mm	26 (b)	32 (d)	aLWT > 20.5 mm	140 (b)	10 (d)
Number Needed to Treat = 75			Number Needed to Treat = 64		

Number Needed to Treat = 1/absolute risk reduction. Absolute risk reduction = d/(b + d) − c/(a + c).

## Discussion

TFF stability was reported to be partly predictable by aLWT with a 20.5 mm threshold, which has been incorporated in the preoperative treatment decision^6–8,10^. However, an absolute threshold ignores anatomical differences, which can result in an inaccurate evaluation of stability and subsequently in a higher rate of sLWF. This study established an individualized measure of rLWT for assessment of TFF stability. The feasibility of measuring rLWT using the contralateral femur was demonstrated. Furthermore, a threshold of rLWT = 50.5% was demonstrated to be a more accurate sLWF predictor after DHS implantation compared with the standard aLWT.

Anatomical variation in the proximal femur is known to have an influence on fracture risk. With increased femoral dimensions, such as femoral neck length and thickness, a rising risk of TFF was reported^16,17^. Contrarily, the risk in primary TFF fractures is not affected by CCD^16,17^. However, as shown in part one of this study, CCD correlates with TTT. Therefore, CDD indirectly influences rLWT and accordingly the stability prediction for osteosynthesis. Also, the direct influence of postoperative CCD on stability has previously been demonstrated^5^. The standard aLWT measure is evaluated at a fixed 135° angle with an absolute threshold of 20.5 mm, thus not considering anatomical variation^6,10^. The novel rLWT incorporates this variability by measuring the ratio between LWT and TTT under consideration of an individual CCD, resulting in a patient-specific value and a more accurate measure. Moreover, the 135° angle used in the definition of aLWT was based on the corresponding angle of DHS. TFF might require an implant with a different angle to gain a more anatomical fracture reduction. This raises the question whether or not aLWT is suitable in cases where an implant with a different angle is used. rLWT incorporates the angle variation and is therefore independent of the implant angle, which makes rLWT more suitable in such cases.

Stability is crucial in fracture management. Fracture morphology, defining fracture stability, therefore determines the treatment. Fracture classification systems help orthopaedic trauma surgeons to predict fracture stability based on fracture morphology^10^. Tawari et al. summarized the following unstable fracture configurations in TFF: fractures with posteromedial comminution, reverse oblique fractures, fractures with subtrochanteric extension, avulsed greater trochanter and lateral wall fractures^12^. The lateral wall importance was discussed by Gotfried et al. in understanding the fracture collapse after DHS implantation^7^. After validation in multiple studies, lateral wall thickness was generally accepted as a stability factor for TFF^6, 8–10,12,14^. The lateral wall acts as a buttress during fracture compression allowed by the dynamic characteristics of the DHS and other dynamic implants creating stability. In case of sLWF, extramedullary implants cannot replace the loss in stability, while intramedullary implants medialize the load transmission and the proximal end of the nail is at the level of the greater trochanter, thus providing additional stability^5,10,14,22^. Moreover, lateral wall weakening during surgery is associated with the drillhole of the implant and may result in sLWF, converting TFF into an unstable AO/OTA 31-A3 or subtrochanteric fracture, risking excessive telescoping and collapse^5,7,9^. Subsequently, the collapse contributes to postoperative morbidity, disability and the need for reoperation^7,10,14^.

This study investigated whether an adapted, patient-specific rLWT measure could allow for higher sensitivity and specificity in sLWF prediction compared to the current standard aLWT. The results revealed increased sensitivity and specificity (by 1.0% and 3.5%, respectively) for rLWT (Fig. 2). The predicted rLWT threshold was 52.5% based on the 20.5 mm aLWT threshold. However, the rLWT threshold, determined by maximizing sensitivity and specificity, was 50.5%, suggesting that the 20.5 mm aLWT threshold is less accurate. Moreover, rLWT was found to be relevant for both AO/OTA 31-A1 and AO/OTA 31-A2 fractures, demonstrating better differentiation compared to aLWT that reached significance only for AO/OTA 31-A2 fractures^6^. Both of these improvements render rLWT a promising candidate for sLWF prediction.

sLWF is associated with an incidence of 20–30% and has a major impact on the patient and the health system requiring revision surgery in 22–45% of cases^6,8,9^. Moreover, sLWF without the need or wish for a reoperation presumably contributes to morbidity, disability, longer rehabilitation and/or mortality. Preventing sLWF is expected to lead to better patient outcome and reduced healthcare costs.

This study has several limitations. First, the proposed measuring protocol relies on the presence of a healthy, non-deformed contralateral femur or a previous pelvic radiograph. aLWT can be a good alternative in cases where this data is not available. Second, TTT is a newly proposed measure that has not yet been validated in other studies. Third, the measuring protocol described in this study relies on a two-dimensional radiograph, which is challenging for measurement of a three-dimensional fracture line in an externally rotated femur. However, the same applies to the already established aLWT measure. Evaluating rLWT in a three-dimensional aspect from a computed tomography (CT) scan should be more accurate but would come at a higher cost and radiation exposure^23,24^. Today's standard in diagnosing TFF does not imply a CT scan and that is why the latter cannot be used for rLWT definition^25,26^. Third, the evaluation has been performed retrospectively. Further prospective clinical studies are needed to confirm its proposed accuracy.

The findings of this study suggest that rLWT should be favored over the aLWT as it achieves improved accuracy in predicting sLWF. The high incidence of TFF will continue to increase with the aging population^2,3^. Although rLWT has not yet been used in clinical practice, this study demonstrates increased accuracy compared with aLWT based on the same dataset. The obtained increase in specificity of sLWF prediction by 3.5% via rLWT corresponds to a Number Needed to Treat of 64 patients for preventing one more sLWF case, which has an impact on TFF treatment. Therefore, the slightly decreased reproducibility and increased complexity of the rLWT versus aLWT measurement should not be limiting factors. rLWT with the 50.5% stability threshold could be used for implant selection; however, future studies are needed to investigate this aspect. Moreover, implant selection depends on multiple factors and should be evaluated by the surgeon per individual case.

## Conclusion

The novel rLWT is a more accurate and individualized predictor of sLWF after DHS fixation compared withto the standard aLWT. The 50.5% rLWT threshold could be used as an indicator for implant selection, with more sLWF and re-operations expected in extramedullary DHS treatment when the rLWT is lower than 50.5%. The current findings indicate that DHS should not be used with a rLWT below 50.5% but future studies will be needed to investigate the aspect of implant selection.

## Data Availability

Data are available from the authors upon reasonable request by contacting the corresponding author (Kenneth P. van Knegsel).
